# Cladding Mode Fitting-Assisted Automatic Refractive Index Demodulation Optical Fiber Sensor Probe Based on Tilted Fiber Bragg Grating and SPR

**DOI:** 10.3390/s22083032

**Published:** 2022-04-15

**Authors:** Wenwei Lin, Weiying Huang, Yingying Liu, Xiaoyong Chen, Hang Qu, Xuehao Hu

**Affiliations:** 1 Research Center for Advanced Optics and Photoelectronics, Department of Physics, College of Science, Shantou University, Shantou 515063, China; wwlin@stu.edu.cn (W.L.); 19yyliu@stu.edu.cn (Y.L.); haqux@stu.edu.cn (H.Q.); 2 Key Laboratory of Intelligent Manufacturing Technology of MOE, Shantou University, Shantou 515063, China; 3 Guangdong Provincial Key Laboratory of Digital, Signal and Image Processing, Department of Electronic and Information Engineering, College of Engineering, Shantou University, Shantou 515063, China; 19wyhuang@stu.edu.cn; 4 School of Electrical Engineering and Intelligentization, Dongguan University of Technology, Dongguan 523808, China; xychen@dgut.edu.cn

**Keywords:** tilted fiber Bragg grating, surface plasmon resonance, surrounding refractive index, cladding mode, automation

## Abstract

In the paper based on surface plasmon resonance (SPR) in a tilted fiber Bragg grating (TFBG), a novel algorithm is proposed, which facilitates demodulation of surrounding refractive index (SRI) via cladding mode interrogation and accelerates calibration and measurement of SRI. Refractive indices with a tiny index step of 2.2 × 10^−5^ are prepared by the dilution of glucose aqueous solution for the test and the calibration of this fiber sensor probe. To accelerate the calibration process, automatic selection of the most sensitive cladding mode is demonstrated. First, peaks of transmitted spectrum are identified and numbered. Then, sensitivities of several potentially sensitive cladding modes in amplitude adjacent to the left of the SPR area are calculated and compared. After that, we focus on the amplitudes of the cladding modes as a function of a SRI, and the highest sensitivity of −6887 dB/RIU (refractive index unit) is obtained with a scanning time of 15.77 s in the range from 1520 nm to 1620 nm. To accelerate the scanning speed of the optical spectrum analyzer (OSA), the wavelength resolution is reduced from 0.028 nm to 0.07 nm, 0.14 nm, and 0.28 nm, and consequently the scanning time is shortened to 6.31 s, 3.15 s, and 1.58 s, respectively. However, compared to 0.028 nm, the SRI sensitivity for 0.07 nm, 0.14 nm, and 0.28 nm is reduced to −5685 dB/RIU (17.5% less), −5415 dB/RIU (21.4% less), and −4359 dB/RIU (36.7% less), respectively. Thanks to the calculation of parabolic equation and weighted Gauss fitting based on the original data, the sensitivity is improved to −6332 dB/RIU and −6721 dB/RIU, respectively, for 0.07 nm, and the sensitivity is increased to −5850 dB/RIU and −6228 dB/RIU, respectively, for 0.14 nm.

## 1. Introduction

Refractive index measurements are required for a variety of applications, such as food/beverage quality control, oil production monitoring, cosmetic and pharmaceutic industries, etc., which normally need minimally invasive and remote interrogation devices. In recent decades, fiber-optic refractive index sensors have been widely used in both academic and industrial fields. To measure refractive indices surrounding a fiber probe, evanescent field of fiber-guided modes typically has to penetrate into the analytes [1]. This mechanism is demonstrated in grating-based fiber devices [2]. Among them, TFBGs are generally short period (pitch: ~500 nm) gratings with refractive index modulations angled (θ < 45°) with respect to the fiber transverse plane with both core mode coupling and core-cladding mode couplings. For the latter, the amplitude and the central wavelength of the cladding mode resonance vary as a function of SRI due to their influences on the phase velocities or the effective refractive indices of the cladding modes [3]. When the SRI reaches the effective refractive index of a cladding mode, the latter becomes no longer reflected but radiated at the fiber-surrounding medium interface. Thus, with increasing SRI, the cladding mode resonances could disappear from the left to the right part of a transmitted spectrum.

Based on these properties, Hu et al. computed the area delimited by the upper and lower envelopes of the cladding modes in the TFBG transmitted amplitude spectrum in different SRI conditions from 1.42 to 1.49. However, this method is not accurate for SRI measurement [4]. A simpler demodulation method was reported by Chan et al. to record the evolution of the central wavelengths of the cladding modes with a measurement accuracy ~10^−4^ in the SRI range from 1.25 to 1.43 [5]. Zhou et al. presented a method to calculate the SRI at the exact wavelength positions in the near infrared frequency with an accuracy of ±5 × 10^−5^ by the calibration of the absolute core index dispersion of the TFBG based on multiple cladding mode resonances. However, the method is very complex and requires a semi-analytic simulation tool [6]. In addition, cut-off wavelength where the cladding mode resonance suddenly declines can serve as an indication for SRI measurement. Pham et al. reported a demodulation method based on cut-off wavelength which was identified by calculating the relative transmission spectra and using the threshold value. As the cut-off wavelength shifts from one to another cladding mode resonance when the SRI changes, this method is suitable for large range SRI monitoring, but the accuracy is only ~10^−3^ [7]. Tomyshev et al. applied Fourier filtering to get rid of spectral noise, and then plotted smooth curves through the array of points represented by peak vertices. Following that, the zero of the second derivative of the arctangent was regarded as the cut-off wavelength corresponding to a SRI. The resolution was estimated to 3.7 × 10^−5^ [8].

To further improve the accuracy of the refractometer, a nanoscale gold layer is usually deposited on the surface of a TFBG. Assuming that light propagating in the fiber cladding is radially polarized (so-called P-polarization), SPR is generated at the gold-medium interface, which enhances the sensitivity of the cladding modes adjacent to the SPR area or signature [9]. It was demonstrated that for a 10° TFBG, the narrowband resonance is just below the SPR signature presented wavelength shifts with a resolution better than 10^−5^ RIU and a quality factor of ~10^5^ [9]. Compared to wavelength-based approaches, amplitude-based methods are more advantageous, as changes are much easier to measure by commercial near infrared optical spectrum analyzers (OSAs). Caucheteur et al. demonstrated that the amplitude evolution of the most sensitive cladding mode showed a sensitivity of −3600 dB/RIU for a 6° TFBG-SPR [10]. In 2020, based on the same methodology, Loyez et al. used a functionalized gold film deposited on a 8° TFBG to successfully detect breast cancer cells with a low concentration of 10 cells/mL in vitro by monitoring the sensitive modes closest to the SPR region [11]. In 2016, Caucheteur et al. demonstrated the most sensitive cladding mode resonance in the transmitted amplitude spectrum of a 16-mm-long 37° TFBG by varying the atmospheric pressure with an air refractive index sensitivity of 204 nm/RIU and 5515 dB/RIU. Furthermore, with the acoustic wave and a tunable laser, whose wavelength was positioned on the edge of the most sensitive cladding mode resonance, a limit of detection (LOD) very close to 10^−8^ was obtained [12].

In addition to the monitoring of the single selective cladding mode beside the SPR signature, other demodulation methods based on envelopes are discussed in the following text. Caucheteur et al. reported that the polarization dependent on loss upper envelope was computed to retrieve the minimum value as the SPR wavelength, showing a high SRI sensitivity of 673 nm/RIU and a resolution of slightly better than 10^−5^ [9]. Lobry et al. monitored the fit of the lower envelope of the SPR signature to sense the presence of the breast cancer biomarker of HER2 [13]. Leitão et al. proposed a demodulation method based on tracking the local maximum of the SPR signature of the lower envelope of the TFBG spectrum with a cortisol detection sensitivity of 0.275 nm/ng.mL^−1^ [14]. Similar to Ref. [8], after filtering, Manuylovich et al. calculated the minimum of the fitted upper envelope of the SPR signature. The method allowed refractive index measurements with a resolution of 3 × 10^−6^ RIU [15]. Though single cladding mode and envelope demodulation method are promising for SRI measurements, automatic calibration and SRI measurement have not been investigated before. Additionally, in previous works, the amplitude and the central wavelength of a cladding mode were generally selected artificially, and the wavelength interval (resolution) of the sampling points in the OSA was not discussed, which could bring extra errors for both amplitude and wavelength of a cladding mode.

In the paper, we propose a novel algorithm to demodulate SRI variations automatically by selection and calibration of the most sensitive cladding modes adjacent to the SPR area in a TFBG-SPR sensor probe. The demodulation algorithm contains three steps: (1) peak identification with serial numbers and cladding mode selection, (2) the most sensitive cladding mode selection, and (3) improvement of both the efficiency and the sensitivity for SRI measurement using cladding mode fitting technique. The key novelty lies in (1) dramatic reduce of spectral scanning time (lower wavelength resolution) by cladding mode fitting technique with similar SRI sensitivity, and (2) improvement of SRI sensitivity by weighted Gauss fitting method.

## 2. Experiment

### 2.1. Fabrication of TFBG-SPR Sensor Probe

The fiber used in this work is a single-mode silica fiber (Corning Incorporated), which was hydrogen-loaded with a pressure of 1500 psi at a temperature of 50 °C for one week to improve the core photosensitivity before grating inscription. Afterwards, the TFBG was photo-inscribed using the ultraviolet laser phase mask technique [16]. A cylindrical lens was positioned in front of the phase mask to enhance the pulse power density focused on the fiber core. With a rotation of the phase mask, a ~2-cm-long TFBG with a tilt angle of 18° was obtained by spatially scanning the beam along the fiber axis [17]. Then, the TFBG was gold-coated using a sputtering technique and double deposition technique [18]. The average thickness of the gold layer was ~40 nm, estimated by the growth rate of a reference film thickness. Although the spectral evolution of the TFBG-based sensor is usually recorded in transmission with both ends of the TFBG connected to a broadband source (BBS) and a OSA, respectively, it is difficult for the sensor probe to be functionalized for in-situ detection. Thus, in this work, an additional gold coating with a thickness of ~500 nm was deposited on the end-face of the fiber located several mm from the TFBG as a highly reflective broadband mirror, which allows interrogating the optical spectra of the sensor probe in reflection [19].

### 2.2. SRI Measurement in Glucose Aqueous Solutions

**Figure 1:**
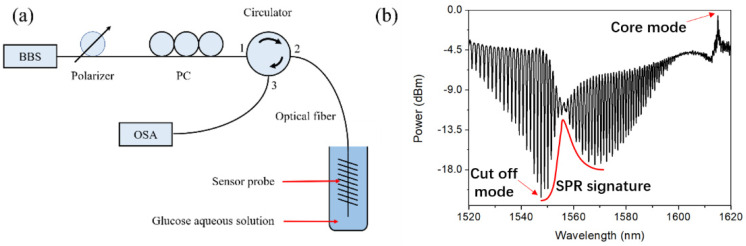
(**a**) Schematic set-up of the TFBG-SPR sensor probe for the SRI measurement in glucose aqueous solutions, and 1, 2 and 3 represent the port number of the circulator; (**b**) P-polarized reflected spectrum of the TFBG-SPR sensor probe in the glucose aqueous solution with a SRI of ~1.3573.

**Figure 2:**
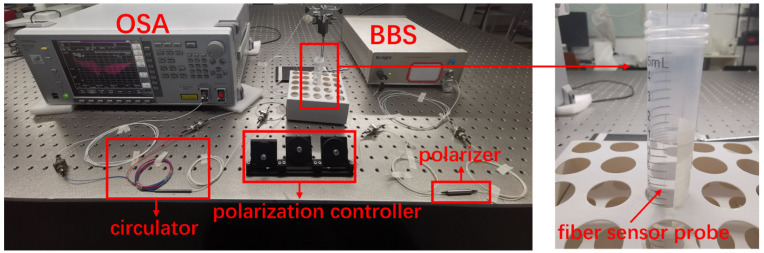
Real experimental set-up for SRI sensing.

The experimental setup for SRI measurement is composed of a BBS (Golight, ASE C+L LIGHT SOURCE, Shenzhen, China), a polarizer, a polarization controller, a circulator, a fiber sensor probe, and a OSA (Anritsu, MS9740A, Atsugi, Japan), as shown in Figure 1a. The input state of polarization was adjusted to generate a P-polarized spectrum with a SPR signature. The SRI measurement was conducted by immersing the probe in glucose aqueous solutions (refractive index ~1.3573) with small refractive index changes of the order of ~10^−5^. The reflected spectrum of the TFBG in P-polarization to investigate in the range from 1520 nm to 1620 nm is shown in Figure 1b with a SPR signature situated at ~1557 nm. The peak situated rightmost in this figure is pertinent to the core mode, which could be used for temperature compensation, while the comb-like spectral fluctuations situated to the left of the core-mode reflection peak is due to the couplings between the core mode and the cladding modes. The real experimental set-up is displayed in Figure 2.

It is worth mentioning that the refractive index change ~10^−5^ in the experiment is an estimated value, as the Abbe refractometer used in this work has a resolution of 1 × 10^−4^. This solution refractive index estimation was conducted in the following steps. First, 20 g glucose was added into 100 mL deionized water at room temperature, and the refractive index was measured to be 1.3574. Second, a couple of drops of 10 μL deionized water were added into the glucose aqueous solution prepared in the first step until the measured refractive index was reduced to 1.3573. Then, 10 mL of the solution obtained in step two was transferred into a test tube. Afterwards, four drops of 10 μL of deionized water were successively added to the solution without refractive index change measured by the Abbe refractometer. However, with one more drop, the refractive index decreased to 1.3572. Thus, five solutions with different refractive index ~1.3573 were prepared, and the refractive index difference induced by one drop was estimated to be (2.2 ± 0.3) × 10^−5^. In the following text, the glucose aqueous solution diluted by four drops is regarded as the initial solution with the lowest refractive index ~1.3573. The linearity of the refractive index changes, as a function of drop of 10 μL deionized water was confirmed by another experiment covering a larger refractive range of 1.3570–1.3578.

## 3. SRI Demodulation Method

**Figure 3:**
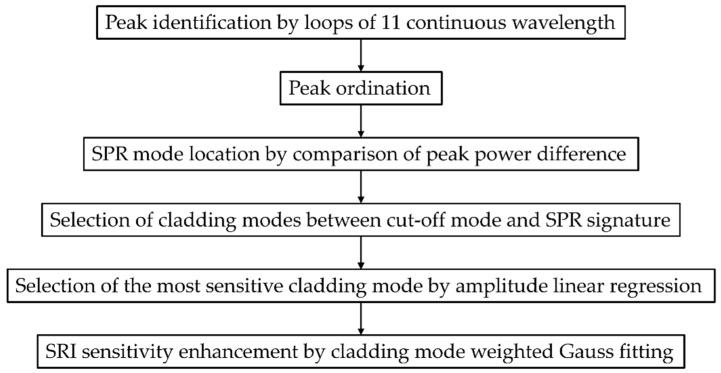
Schematic of SRI demodulation algorithm.

A schematic of SRI demodulation algorithm is demonstrated in Figure 3.

### 3.1. Peak Identification and Cladding Mode Selection

**Figure 4:**
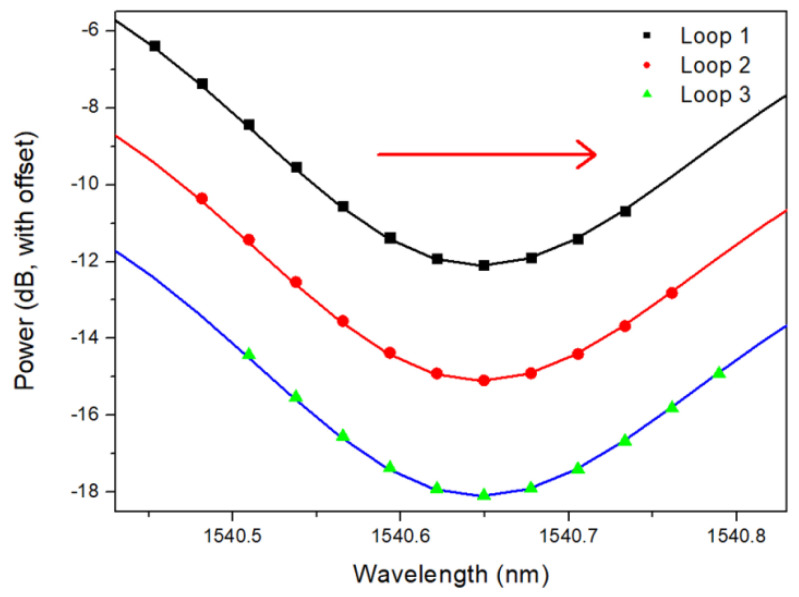
Loops of 11 continuous wavelengths around the peak ~1540.6 nm in the initial glucose aqueous solution with a SRI of ~1.3573.

**Figure 5:**
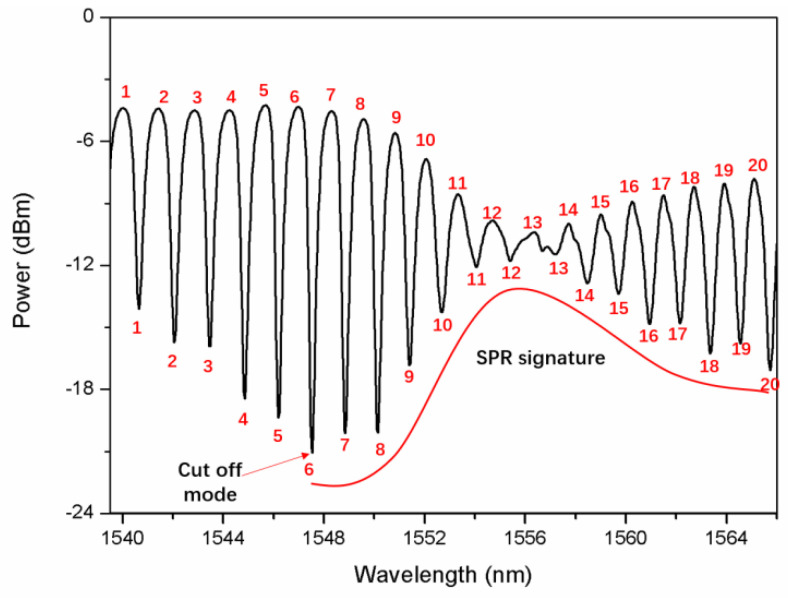
Max-peaks and min-peaks with serial numbers, such as 1, 2, 3, etc., in the initial glucose aqueous solution with a SRI of ~1.3573.

The peak identification was conducted in the wavelength range from 1539.5 nm to 1566 nm with a wavelength resolution of 0.028 nm, including 20 cladding mode resonances adjacent to the SPR signature. Due to the small refractive index change, the SPR signature almost remained during this experiment. Driven by the wish to calibrate this sensor probe automatically, first of all, the peak detection was conducted by comparing transmitted power of 11 continuous wavelength sampling points from 1539.5 nm, which looped to 1566 nm in turn [20,21,22,23]. The amount 11 of the wavelengths in one sequence is optimized to better filter out the spectral noise. Figure 4 presents an example of three loops around the peak ~1540.6 nm. If the power of the 6th wavelength in the middle of this sequence is the maximum or the minimum, the 6th wavelength is regarded as the peak, so-called max-peak or min-peak. Therefore, the 6th wavelength in loop 3 is regarded as the central wavelength of the min-peak (cladding mode resonance). Using the same methodology, each peak was issued a serial number in order, as shown in Figure 5.

After peak ordination, the SPR signature is located by the following two steps. First, power differences between max-peaks and min-peaks with the same serial numbers were calculated. Second, by comparison the minimal power difference was obtained, which belongs to the SPR mode with serial number 13. The area around the SPR mode is defined as the SPR signature [8,15]. Meanwhile, the cut-off mode located to the blue side of the SPR mode was identified by searching the lowest amplitude of the cladding mode, which has a serial number of 6. Thus, the cladding modes of most interest (No. 7–No. 12), sandwiched between the cut-off mode and the SPR signature, were adopted for the SRI measurement, as illustrated in Figure 5.

### 3.2. Selection of the Most Sensitive Cladding Mode

**Figure 6:**
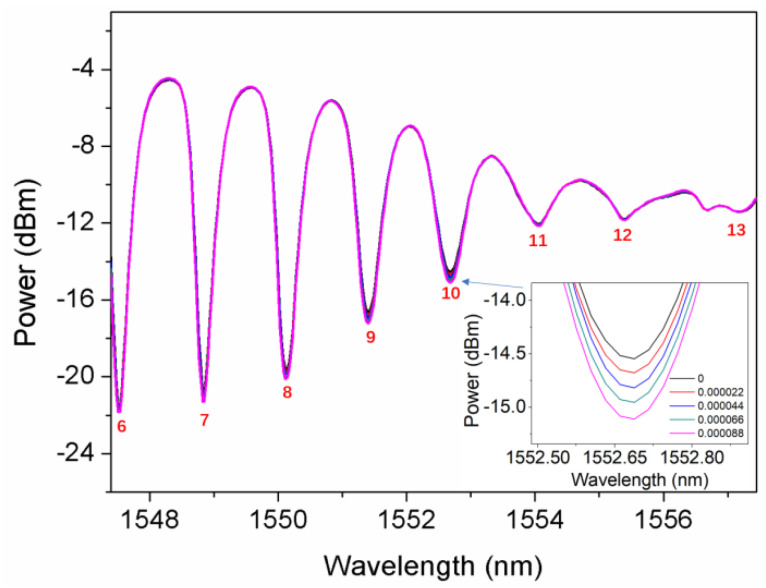
Spectra adjacent to the SPR signature in different glucose aqueous solutions (refractive index ~1.3573) with a SRI change of 8.8 × 10^−5^; inset: zoomed-in spectra of the most sensitive cladding mode No. 10.

**Figure 7:**
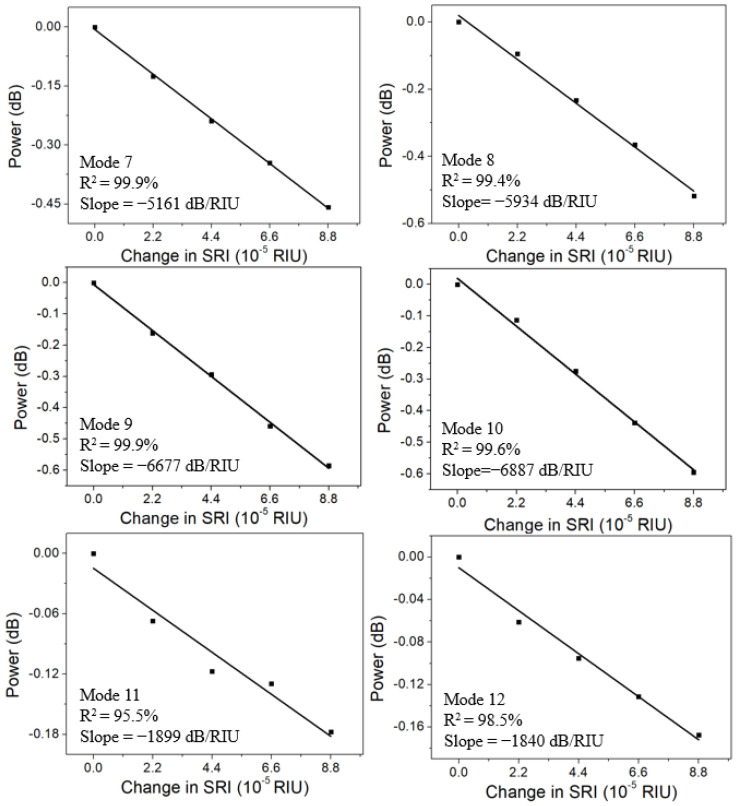
Linear regressions of the amplitudes of the cladding modes No. 7–No. 12 in different glucose aqueous solutions (refractive index ~1.3573) with a SRI change of 8.8 × 10^−5^.

Figure 6 shows the spectra adjacent to the SPR signature in different glucose aqueous solutions (refractive index ~1.3573) with a SRI change of 8.8 × 10^−5^. Due to small refractive index variation, only amplitudes of the cladding modes were investigated. The linear regressions of the amplitudes of the sensitive cladding modes (No. 7–No. 12) are demonstrated in Figure 7. The linear regression equation [24,25] is validated to build the relationship between the independent variables (SRI) and the dependent variables (amplitude) for the prediction of SRI according to the amplitude of the cladding mode. It is of great importance that the factor of $R^{2}$ [26] is used to evaluate whether both independent and dependent variables in the regression equation are a good fit, defined by

(1) $$R^{2}=1-\frac{\sum {\left(y_{i}-{\hat{y}}_{i}\right)}^{2}}{\sum {\left(y_{i}-\bar{y}\right)}^{2}}$$

where $y_{i}$ is the measured value, ${\hat{y}}_{i}$ is the data value after fitting, and $\bar{y}$ is the average measured value. The subscript i represents the ordinal of the data.

To avoid the downside, only the linear regressions for the cladding modes (No. 7–No. 10) with $R^{2}$ higher than 99% showing good linearity were considered. After comparison, the most sensitive cladding mode No. 10 with a sensitivity of −6887 dB/RIU was obtained, whose spectral variation is presented in the inset of Figure 6. On the contrary, the linear regressions for cladding modes No. 11 and No. 12 with lower $R^{2}$, which are 95.5% and 98.5%, respectively, were abandoned. Meanwhile, they present the lowest sensitivities of −1899 dB/RIU and −1840 dB/RIU, respectively.

### 3.3. Cladding Mode Fitting for Efficiency and Sensitivity Improvement

**Figure 8:**
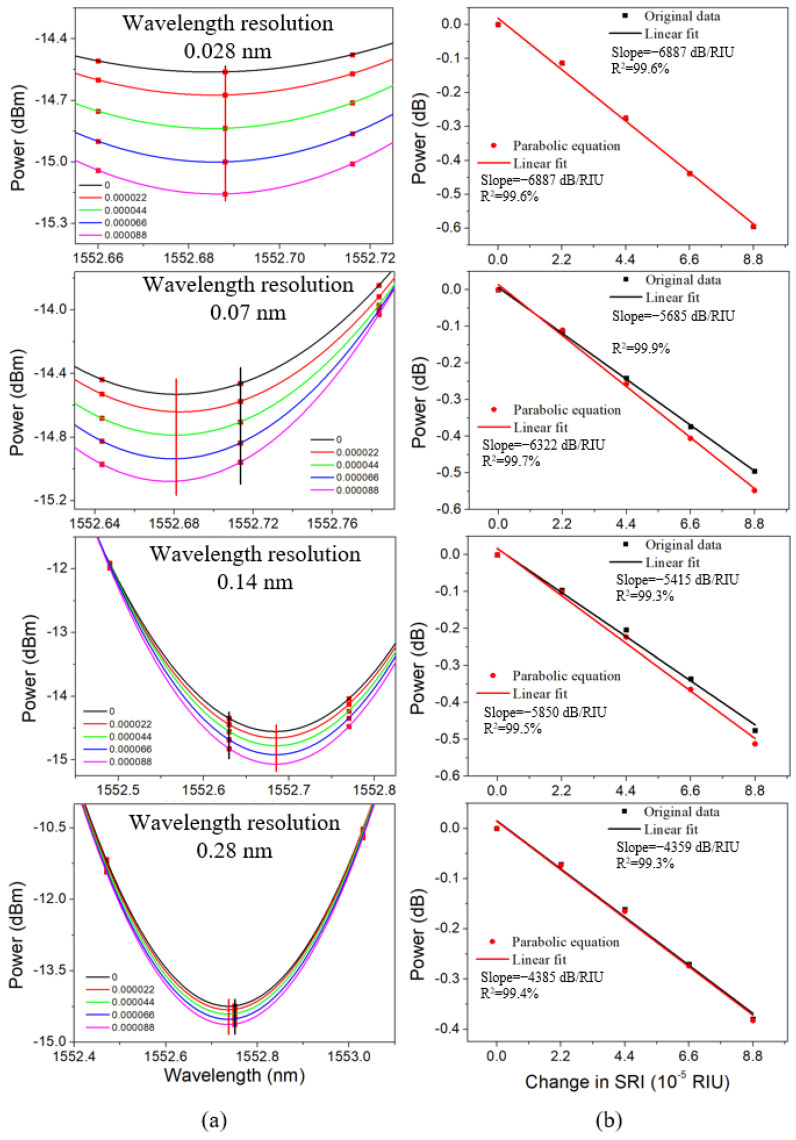
Transmitted amplitude spectra of the cladding mode No. 10 (**a**) and linear fit of the cladding mode amplitude (**b**) with three original data and parabola calculated based on these data, respectively, in different glucose aqueous solutions (refractive index ~1.3573).

Driven by the demand of biochemical reaction process monitoring in real time, fast SRI demodulation technique is desired. Here, although the highest sensitivity of −6887 dB/RIU for Mode 10 was obtained, it took 15.77 s to scan the wavelength range from 1520 nm to 1620 nm, which was not fast enough for real-time measurement. To improve that, the wavelength resolution was decreased from 0.028 nm to 0.07 nm, 0.14 nm, and 0.28 nm, respectively, and the scanning time was shortened to 6.31 s, 3.15 s, and 1.58 s, accordingly. The transmitted amplitude spectra and the linear fits of the cladding mode amplitudes (vertical line in black) are shown in Figure 8. It is found that the SRI sensitivity gradually decreases from −6887 dB/RIU to −5685 dB/RIU (17.5% less), −5415 dB/RIU (21.4% less), and −4359 dB/RIU (36.7% less), respectively. Thus, there is a trade-off between scanning time and refractometric sensitivity. Thanks to the calculation of parabolic equation based on three original data centered on the wavelength corresponding to the cladding mode amplitude (square point in red), the amended amplitude of the cladding mode (vertical line in red) was obtained. For 0.028 nm, the sensitivity remained, which could be attributed to the coincidence of the cladding mode amplitudes before and after the calculation of parabolic equation, and the high wavelength resolution. Nevertheless, the sensor probes with lower wavelength resolutions and faster scanning rates are more attractive to investigate. It is discovered that the improved sensitivities, i.e., −6322 dB/RIU (11.2% improvement) and −5850 dB/RIU (8.0% improvement), were obtained for the resolution of 0.07 nm and 0.14 nm, respectively. For the wavelength resolution of 0.28 nm, the sensitivity is hardly improved due to the lack of data points.

Afterwards, odd number over three of original data centered on the cladding mode were considered for Gauss fitting. In addition, since the data centered on the min-peak of the cladding modes play more important roles for Gauss fitting, the weighting rule that data closer to the min-peak have greater weight, and vice versa, is applied for Gauss fitting improvement. Weighted fitting has been applied successfully in industry and pharmacy in previous works [27,28,29]. The specific weight formula is as follows:

(2) $$w\left(i\right)={\left[1-\frac{\left|x_{i}-x_{min}\right|}{x_{min}-x_{L}}\right]}^{4}\;x_{L}\leq x_{i}\leq x_{min}$$

(3) $$w\left(i\right)={\left[1-\frac{\left|x_{i}-x_{min}\right|}{x_{R}-x_{min}}\right]}^{4}\;x_{min}<x_{i}\leq x_{R}$$

where x represent the wavelength, the subscript min, L and R denote the wavelength corresponding to the min-peak, the leftmost and the rightmost data, respectively, and w(i) represents the weight. Therefore, the $R^{2}$ is adapted to be

(4) $$R^{2}=1-\frac{\sum w\left(i\right){\left(y_{i}-{\hat{y}}_{i}\right)}^{2}}{\sum {\left(y_{i}-\bar{y}\right)}^{2}}$$

**Figure 9:**
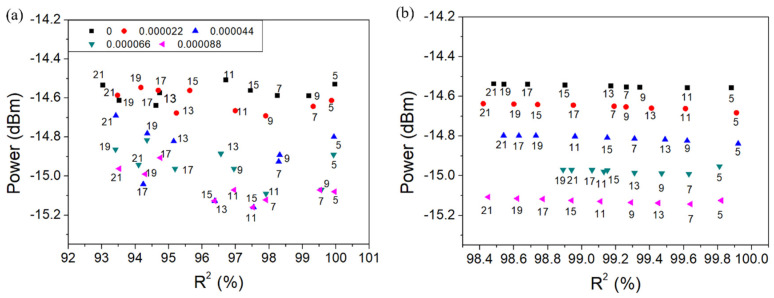
Amplitude of the cladding mode No. 10 after Gauss fitting in terms of SRI change 8.8 ×10^−5^ for wavelength resolution 0.07 nm (unweighted (**a**) and weighted (**b**)).

**Figure 10:**
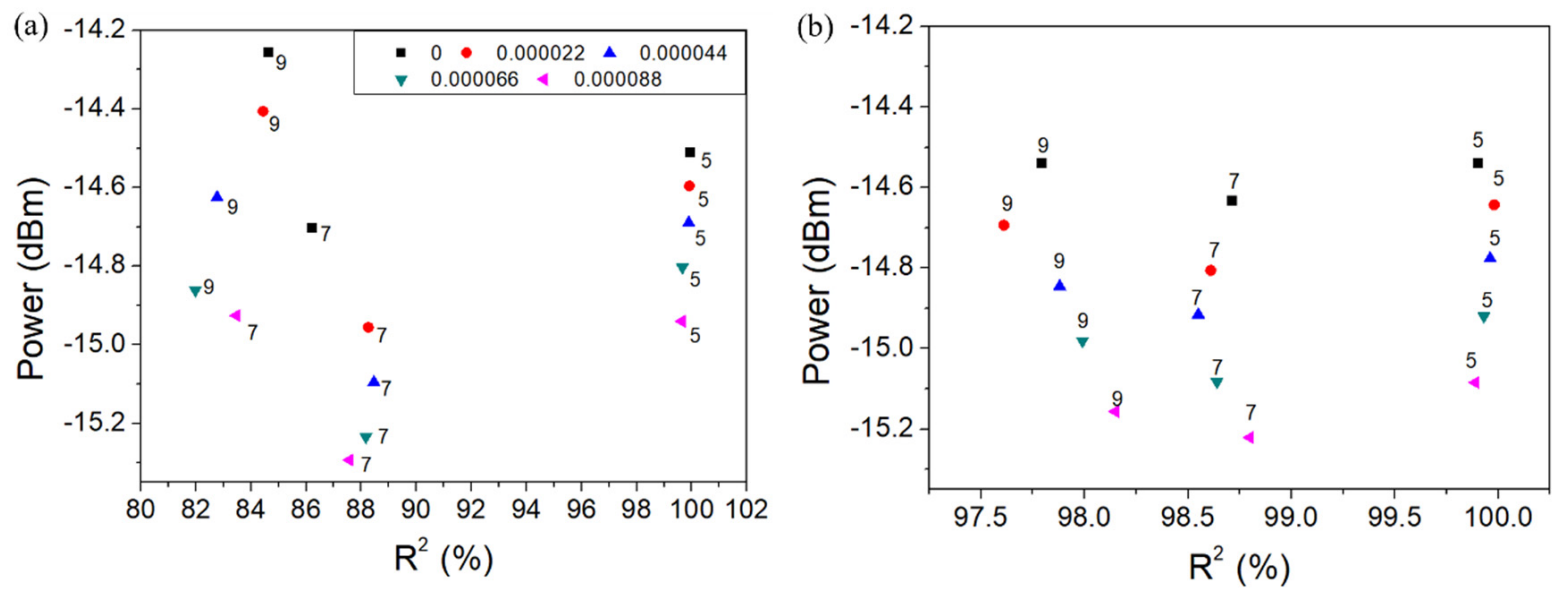
Amplitude of the cladding mode No. 10 after Gauss fitting in terms of SRI change 8.8 × 10^−5^ for wavelength resolution 0.14 nm (unweighted (**a**) and weighted (**b**)).

The amplitude of the cladding mode No. 10 after unweighted and weighted Gauss fitting and the amount of original data used for fitting as a function of $R^{2}$ are shown in Figure 9 and Figure 10 for wavelength resolution 0.07 nm and 0.14 nm, respectively, in terms of SRI change 8.8 × 10^−5^. Then, after the calculation of the average of the cladding mode amplitudes resulting from Gauss fitting ($R^{2}$ greater than 99%), a linear fit between the average amplitude and the SRI change was conducted.

**Figure 11:**
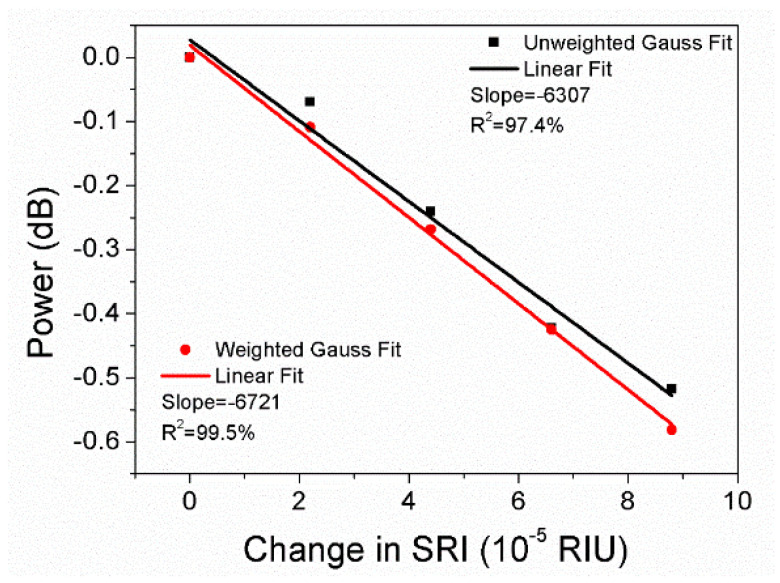
Linear regression of the average cladding mode amplitude obtained by unweighted and weighted Gauss fitting in terms of SRI change 8.8 × 10^−5^ for the wavelength resolution 0.07 nm.

For the wavelength resolution 0.07 nm, the sensitivities obtained by unweighted and weighted Gauss fitting are −6307 dB/RIU and −6721 dB/RIU, respectively, as shown in Figure 11, which are higher than the sensitivity resulting from the original data −5685 dB/RIU. Additionally, the sensitivity calculated from unweighted Gauss fitting is close to −6332 dB/RIU resulting from the parabolic equation, and the linear fit $R^{2}$ corresponding to unweighted Gauss fitting is only 97.4%, which is inappropriate for sensing applications. Compared to unweighted Gauss fitting, the sensitivity obtained by weighted Gauss fitting is improved by 6.6%, and the linear fit $R^{2}$ is 99.5%. Both the sensitivity and the $R^{2}$ are similar to the results obtained from original data or parabolic equation for the wavelength resolution 0.028 nm.

**Figure 12:**
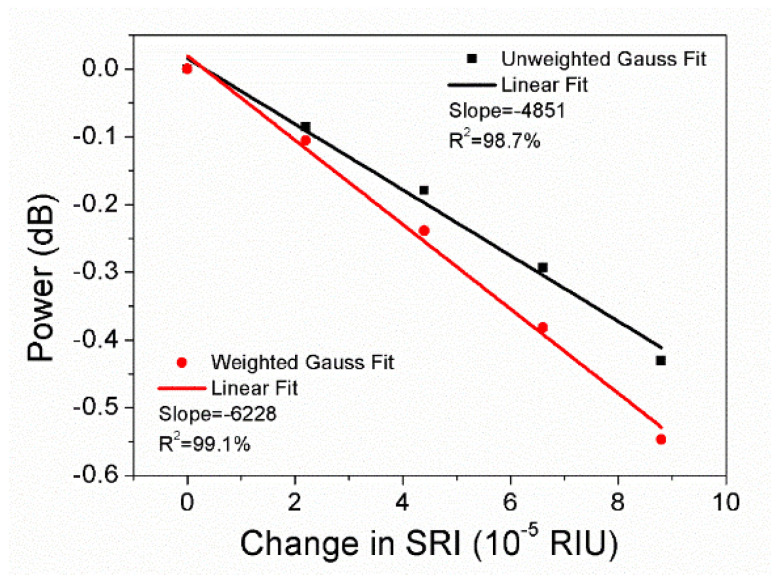
Linear regression of the cladding mode amplitude obtained by 5-data unweighted and weighted Gauss fitting in terms of SRI change 8.8 × 10^−5^ for the wavelength resolution 0.14 nm.

**Figure 13:**
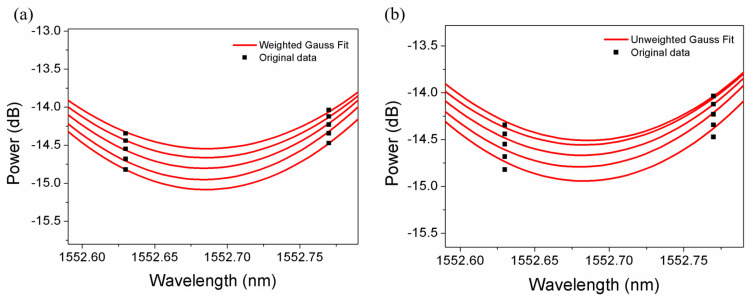
Transmitted spectra of the cladding mode No. 10 with weighted (**a**) and unweighted (**b**) Gauss fitting in terms of SRI change 8.8 × 10^−5^ for the wavelength resolution 0.14 nm.

For the wavelength resolution 0.14 nm, the sensitivities obtained by 5-data unweighted and weighted Gauss fitting are −4851 dB/RIU and −6228 dB/RIU, respectively, as depicted in Figure 12. It is found that compared to unweighted Gauss fitting, the sensitivity calculated from weighted gaussian fitting is improved by 28.4% with the linear fit $R^{2}$ improved from 98.7% to 99.1%. The sensitivity obtained is higher than −5415 dB/RIU from original data and −5850 dB/RIU from parabolic equation. Although the sensitivity (calculated from weighted Gauss fitting) of −6228 dB/RIU for the wavelength resolution 0.14 nm is 7.3% smaller than −6721 dB/RIU for the wavelength resolution 0.07 nm, the scanning time of the OSA is reduced by 50%. The spectra of the cladding mode No. 10 with unweighted and weighted Gauss fitting, respectively, are illustrated in Figure 13, showing a better fit at the bottom after weighted Gauss fitting. A comparison of the slope and $R^{2}$ after linear regression of the amplitude of the cladding mode No. 10, resulting from original data, parabolic equation, unweighted and weighted gauss fit, is presented in Table 1.

It is worth mentioning that both the SRI and the fiber refractive index could be influenced by the temperature, so the automatic calibration process for small refractive index change sensing could be interfered. Thus, in this work the calibration process was conducted rapidly in the temperature-controlled environment, and the temperature influence was consequently omitted.

## 4. Conclusions

In summary, we proposed a new algorithm to automatically demodulate SRI changes by selecting and calibrating the most sensitive cladding mode in the adjacent SPR region of the TFBG-SPR sensor probe. After that, both the calculation of parabolic equation, unweighted, and weighted Gauss fitting based on the original data were implemented for the improvement of the efficiency and the sensitivity of the TFBG-SPR sensor probe, which paves the way to the automatic calibration and ease of application, and may potentially be integrated into a commercial device in the future.

## Figures and Tables

**Table 1:** Comparison of the slope and $R^{2}$ after linear regression of the amplitude of the cladding mode No. 10 resulting from original data, parabolic equation, unweighted, and weighted gauss fit.

Wavelength Resolution		Original Data	Parabolic Equation	Unweighted Gauss Fit	Weighted Gauss Fit
0.07 nm	Slope (dB/RIU)	−5685	−6332	−6307	−6721
	$R^{2}$ (%)	99.9	99.7	97.4	99.5
0.14 nm	Slope (dB/RIU)	−5415	−5850	−4851	−6228
	$R^{2}$ (%)	99.3	99.5	98.7	99.1
